# Shifting Developmental Trajectories During Critical Periods of Brain Formation

**DOI:** 10.3389/fncel.2020.00283

**Published:** 2020-09-10

**Authors:** Nathalie Dehorter, Isabel Del Pino

**Affiliations:** ^1^Eccles Institute of Neuroscience, The John Curtin School of Medical Research, Australian National University, Canberra, ACT, Australia; ^2^Principe Felipe Research Center (Centro de Investigación Principe Felipe, CIPF), Valencia, Spain

**Keywords:** development, plasticity, critical period, neurodevelopmental disorders, brain organoids

## Abstract

Critical periods of brain development are epochs of heightened plasticity driven by environmental influence necessary for normal brain function. Recent studies are beginning to shed light on the possibility that timely interventions during critical periods hold potential to reorient abnormal developmental trajectories in animal models of neurological and neuropsychiatric disorders. In this review, we re-examine the criteria defining critical periods, highlighting the recently discovered mechanisms of developmental plasticity in health and disease. In addition, we touch upon technological improvements for modeling critical periods in human-derived neural networks *in vitro*. These scientific advances associated with the use of developmental manipulations in the immature brain of animal models are the basic preclinical systems that will allow the future translatability of timely interventions into clinical applications for neurodevelopmental disorders such as intellectual disability, autism spectrum disorders (ASD) and schizophrenia.

## Introduction

Normal brain function results from a conserved sequence of developmental processes of cell division, migration, network formation and maturation, directed by intrinsic genetic programs as well as by environmental and systemic cues, extrinsic to the nervous system. Within this sequence, appropriate stimuli induce events of heightened plasticity that are required to develop a given function. This capacity of the brain to reorganize during unique developmental time windows in adaptation to the environment has been termed “critical period.”

In the form of electrical activity, experience during development drives the structural and functional organization of neural connectivity (for review Khazipov and Luhmann, 2006). The best studied examples have been the critical periods for sensory functions such as ocular dominance (OD) in the visual system. During this period, monocular deprivation induces an OD shift in the visual system (for review Hensch, 2004; Levelt and Hübener, 2012; Hensch and Quinlan, 2018; Hooks and Chen, 2020). Electrical activity also controls crucial time windows taking place long before the arrival of sensory-driven stimuli. Since early studies of Galli and Maffei (1988), it has been shown that patterns of spontaneous activity originating from the developing retina (for review Ford and Feller, 1995; Feller, 2012) and within local intracortical circuits (for review Siegel et al., 2012) participate to the development of visual circuits (for review Arroyo and Feller, 2016). Spontaneous patterns of activity intrinsic to the cerebral cortex or arriving from subcortical sources have also been characterized in other sensory systems such as the somatosensory and auditory cortex (Allene et al., 2008; Babola et al., 2018; Antón-Bolaños et al., 2019). These early patterns of activity are thought to operate as checkpoints for the correct implementation of the neuronal circuits and consist of intrinsic voltage-gated calcium currents that are followed by non-synaptic and synapse-driven calcium activities (for review Allene and Cossart, 2010). Importantly, they are only observed during specific developmental stages and in a specific sequence (i.e., in the cortex, large plateau assemblies at birth are followed by early network oscillations and giant depolarization potentials) and across regions of the central nervous system such as the spinal cord, the cortex and some subcortical structures (Kirkby et al., 2013; for review Ben-Ari, 2008; Blankenship and Feller, 2010). Previous studies have shown that early spontaneous activity patterns are altered in the neocortex of animal models of neurodevelopmental disorders such as the *Fmr1* knock out model of Fragile X syndrome (FXS; Cheyne et al., 2019) and the *Nr2f1*-deficient model of Bosch-Boonstra-Schaaf optic atrophy syndrome (Del Pino et al., 2020) suggesting a link between the early generation of spontaneous activity and abnormal brain development. Although the specific impact of each pattern of early network activity on adult sensory function remains to be thoroughly investigated, this review summarizes recent findings that bring us closer to answer one major question in developmental neurobiology: how does a timely interaction between molecular programs and electrical activity sculpt neural network formation? Acute manipulations in the developing embryo and in *in vitro* model systems together with transcriptomic data, start to disclose the mechanisms underlying normal and pathological timing of critical periods not only in the sensory and motor systems, but also in associative areas such as the prefrontal cortex (PFC).

Experiences that influence critical periods of plasticity can occur in form of systemic and/or environmental factors of chemical nature such as hormones. Hormones guide behavioral adaptation, adjust the onset of vulnerable time windows and are associated with the transitions in maturational state, including pregnancy, sexual differentiation and puberty (Yamaguchi et al., 2012; for review de Kloet et al., 2005; Piekarski et al., 2017). An important step has been the identification of the tropism of different hormones during developmental transitions. For instance, adequate levels of oxytocin regulate the transient switch of GABA action at birth from excitatory to inhibitory (Tyzio et al., 2006), a transient process necessary for correct brain development. Alterations of this critical period of GABA polarity at birth—transiently excitatory GABA instead of inhibitory—have been associated to the pathophysiology of autism spectrum disorders (ASD) such as Fragile X and Rett syndrome (Tyzio et al., 2014; Fernandez et al., 2019; Lozovaya et al., 2019). Thyroid hormones (THs) are also necessary to maintain normal brain maturation and function (for review see Batista and Hensch, 2019). However, more studies are needed to thoroughly address the specific influence of hormones on critical periods.

Research on experience-dependent developmental plasticity is leading to a better characterization of “critical periods” and “sensitive periods.” Both terms refer to transient time windows during which specific neural circuits undergo a change in response to environmental factors, affecting brain function. However, a careful consideration for what defines and distinguishes these two types of periods of brain development still requires scientific consensus. Therefore, we would like to briefly elaborate on conceptual frameworks and assumptions underlying the assignment of a transient plasticity event as a “critical” or “sensitive period.” Indeed, it was proposed that “critical periods” differ from “sensitive periods” based on dynamics (Knudsen, 2004) i.e., events of gradual plasticity would be classified as “sensitive periods,” while “critical periods” would represent acute shifts during exclusive developmental timepoints in the adaptation of neural systems (White et al., 2013). However, a clear explanation of each type of event based on their common and unique features is still lacking. We revised several definitions proposed in the past (for review Rice and Barone, 2000; Hensch, 2004, 2005; Knudsen, 2004) and reexamined them within the context of the current knowledge.

**Figure 1:**
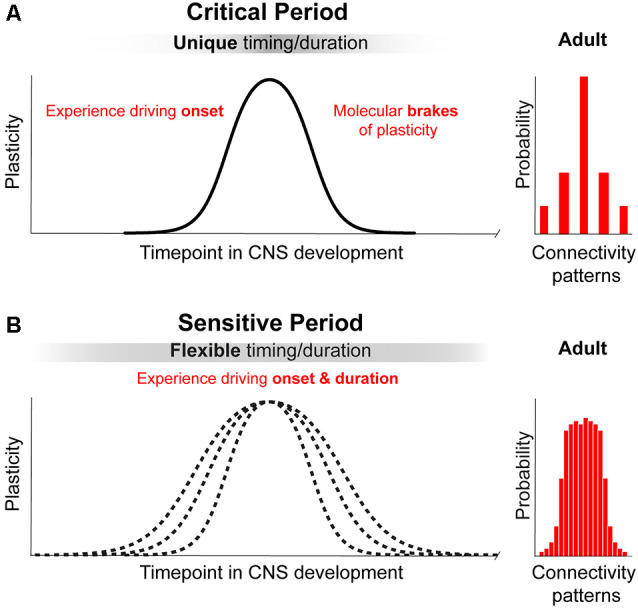
Principles of critical periods compared to sensitive periods of plasticity. **(A)** Critical periods in brain development represent narrow time windows of heightened plasticity driven by environmental input. Closure of critical periods is achieved through molecular brakes that constrain plasticity and allow for permanent structural consolidation of one of a few possible connectivity patterns. **(B)** Sensitive periods in brain development represent broad time windows of gradual change in plasticity driven by environmental input. Sensitive periods are not closed by molecular constrains and can be further reopen by changing environmental cues. The consolidation of one of a broad range of possible patterns of connectivity is reversible and remains functionally dynamic.

We propose that a principle that differentiates a “critical” from a “sensitive” period is the ultimate impact on brain structure and function (for review Knudsen, 2004). During critical periods, experience instructs neural networks to develop into a configuration that cannot be replaced by alternative connectivity patterns (Figure 1, upper panel on the right), leading to irreversible consequences. In other words, a principal feature of a critical period is that it leads to a permanent change necessary for the presence or absence a specific brain function. An example is the input-dependent period for OD, which is necessary for stereopsis and could result in amblyopia (Daw, 1998; for review Hensch and Quinlan, 2018). During sensitive periods, experience leads to many possible network configurations or connectivity patterns that can compensate for each other and are subjected to remodeling during a protracted period of brain development and adulthood (for review Knudsen, 2004; Figure 1). Thus, sensitive periods are characterized by experience-dependent plasticity that is not entirely irreversible and that modulates compensatory connectivity patterns. It tunes the degree of performance of a specific function—within a dynamic and functional range—(e.g., orientation and direction preference (Bachatene et al., 2015), reopen and shift network function to another state as long as enviromental cues are present.

In this review, we focus on distinct types of critical periods of brain development. Critical periods that determine axonal growth and neural circuit organization are named here as “*classical* critical periods,” because their mechanisms and implications for brain function have been investigated for many years. We also feature recent evidence supporting the idea that critical periods occur long before connectivity rearrangements, acting during embryogenesis and early postnatal stages in mice through fate plasticity during neurogenesis and programmed cell death in post-mitotic neurons, respectively. Here, we explain why the latter are emerging as critical periods. Since fate plasticity and programmed apoptosis may endow the cerebral cortex with transient epochs of flexibility to adjust the absolute numbers and type of neurons of cortical networks in response to environmental cues, we propose to term as “*critical periods for network composition*.”

Critical period research has implications beyond a basic understanding of brain formation. It is relevant to continue shedding light on the etiology of neurodevelopmental disorders (Marín, 2016). Here, we brought together recent efforts strengthening the link between transient alterations in brain function and the emergence of symptoms of neurodevelopmental disorders, i.e., intellectual disability, ASD and/or schizophrenia. In addition, we feature preclinical studies that support the notion that critical periods could be successfully employed as windows of opportunity for early therapeutic interventions and shift the pathological time-course of a disease model into a normal or asymptomatic neurodevelopmental trajectory. Finally, we provide with a perspective about recent technological advances that model vulnerable time windows reminiscent of human brain maturation in health and disease, and which represent a promising experimental setting to test most suitable therapeutics in major genetic predispositions to neurodevelopmental disorders.

## Classical Critical Periods and Critical Periods for Network Composition

Classical critical periods of plasticity have been thoroughly characterized in the visual, somatosensory and auditory systems (Barkat et al., 2011; Yang et al., 2012; for review Rice and Barone, 2000; Hensch, 2004, 2005). Studies in animal models have been key to advance in the discovery of molecular mechanisms and plasticity rules that influence the development of sensory systems, as well as their cross-modal regulation (i.e., effect of the function of one brain modality on the function of another).

The maturation of the visual system results from a highly-regulated sequence of events that occurs long before eye-opening and continues to be sculpted at the onset of eye-opening, upon the arrival of visual inputs (for review Daw, 1998; Hooks and Chen, 2007). The shift in OD of binocular neurons induced by monocular deprivation, also known as OD plasticity, has been the classical—probably the most studied—model of critical period plasticity, confined to a vulnerable time window [e.g., from P19 to P32 in mice (Gordon and Stryker, 1996) and P20-P35 in the rat (Fagiolini et al., 1994)]. During this critical time frame, the balance in Excitation/Inhibition (E/I) is adjusted to a final configuration in the adult brain (for review Levelt and Hübener, 2012; Hensch and Quinlan, 2018; Hooks and Chen, 2020). The closure of this period is regulated by molecular brakes halting neural plasticity in the primary visual cortex (Gribizis et al., 2019). Many of these molecular factors initiating and closing critical period plasticity—such as neuromodulatory signals (e.g., acetylcholine), synaptic proteins [e.g., immunoglobulin protein Synaptic Cell Adhesion Molecule 1 (SynCAM1)] and components of the extracellular matrix (e.g., perineuronal nets or PNNs)—are influenced by visual experience and act on main neuronal regulators of critical period in the cerebral cortex i.e., GABAergic interneurons expressing parvalbumin and somatostatin (Fagiolini et al., 2004; Lyckman et al., 2008; Ribic et al., 2019; Yaeger et al., 2019; Yang, 2020; for review Wen et al., 2018).

Some of these molecules are known to play a similar role in critical period plasticity of other sensory areas (McRae et al., 2007; Nakamura et al., 2009; Nowicka et al., 2009). Within the somatosensory system, the whisker system represents a well characterized model of defined sequence of critical periods during postnatal development (Rice and Van der Loos, 1977; for review Erzurumlu and Gaspar, 2012). Parvalbumin interneurons are contributing to activity-dependent changes in the maturation of the somatosensory cortex, during a period spanning from P0 to P14. During this time window, sensory responsiveness and response selectivity to whisker deflections develop in a layer-specific manner within the barrel cortex (van der Bourg et al., 2017). Moreover, sensory deprivation remarkably affects the spatial organization of inhibitory circuits (Lo et al., 2017). In addition to somatosensory input-dependent plasticity—involving NMDA and GABA receptor function and being largely altered by whisker deprivation in the barrel cortex and other relay stations during postnatal stages (Pan et al., 2017)—a recent study reports that spontaneous network activity from thalamic inputs at embryonic stages is also fundamental for somatotopic map development (Antón-Bolaños et al., 2019).

In the primary auditory cortex, a vulnerable period for spectral tuning extends from about P11 to P13 in rat (de Villers-Sidani et al., 2007), followed by another sensitive window, during which the auditory system remains maximally plastic—e.g., P31-P38 in mice (Bhumika et al., 2020), 3.5 years in humans (Sharma et al., 2002). These plastic periods are dependent on the stimulus complexity sculpting the tonotopic organization of the auditory cortex. The environmental influence (or lack of it) in the auditory system has a different impact on the acquisition of hearing depending on the developmental stage. Specifically, hearing loss induced at postnatal day 10 in mice, has greater impact than after sexual maturation (Buran et al., 2014). In the cortex, tonotopic maps are modelled during early life to adapt to the surrounding environment (de Villers-Sidani et al., 2008). The primary auditory cortex is a key hub where neuromodulatory and topographically-organized thalamic inputs meet to tune the cortical layers below. The control of the duration and closure of the vulnerable periods is dependent on the local state of cortical maturation. In particular, inhibitory interneurons in layer 1 (L1) send narrowly descending projections to differentially modulate thalamic drive onto pyramidal and parvalbumin-expressing (PV) cells in L4, creating brief windows of intracolumnar activation. Silencing specific subtypes of L1 interneurons, abolishes map plasticity during the tonotopic critical period (Takesian et al., 2018).

The role of non-neuronal brain cells in critical period plasticity has been less addressed. Oligodendrocyte-neurons interaction through Nogo-66 receptor drive the maturation of intracortical myelination necessary for the closure of OD and auditory plasticity (McGee et al., 2005; Kalish et al., 2020) and might be also a hallmark of critical period conclusion outside sensory modalities. Regions such as the PFC undergo a change in oligodendrocyte maturation and myelination following deprivation of social behavior in mice after weaning (Makinodan et al., 2012, 2016). Of special relevance are single cell RNA sequencing approaches that are accelerating our understanding of the molecular programs implemented by all brain cell types during critical periods. A pioneer effort employed single-cell RNAseq analysis during critical period for tonotopic topography in the primary auditory cortex and provides with a detailed transcriptomic profile of each neuronal and non-neuronal cell type during this critical period for auditory plasticity (Kalish et al., 2020). Following on previous studies describing the role of astrocyte maturation and microglia function in OD plasticity (Müller, 1990; Singh et al., 2016; Sipe et al., 2016), the study of Kalish et al. (2020) substantiates evidences that activation of astrocytes and microglia contribute to critical period plasticity in the neocortex.

The sequence of critical periods of a specific sensory function unfold in coordination with other sensory modalities. The influence of the maturation of one sensory system on another is known as cross-modal or cross-sensory plasticity (for review Morrone, 2010). Initial studies showed that the homeostatic scaling of synaptic plasticity underlies this form of plasticity in sensory areas such as the auditory cortex as well as somatosensory cortices following visual deprivation (Goel et al., 2006; He et al., 2012). Importantly, cross-modal plasticity may also arise outside of the early developmental phase. Plasticity induced by visual deprivation in adult mice results in a potentiation of thalamo-cortical synapses reaching the auditory cortex and leads to an improved processing of the auditory information (Petrus et al., 2014). This indicates that cross-modal plasticity drives the onset of a critical period of synaptic scaling in a brain-region specific manner (i.e., in thalamocortical axons of the auditory cortex, but not of the visual cortex). Further efforts in the study of cross-modal plasticity are fundamental to better understand how critical periods of different brain regions sculpt sensory and high-order brain functions, early in the immature brain (Nardou et al., 2019).

**Figure 2:**
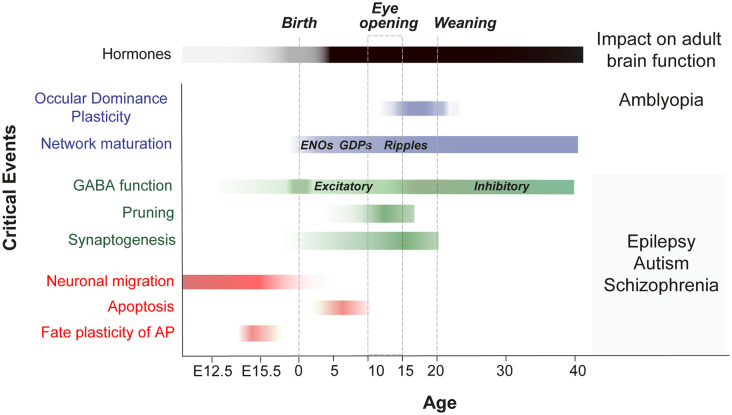
Critical events during embryonic and postnatal periods of brain development that impact adult brain function. Scheme shows a timeline of the key stages in the development of neuronal networks in the mouse brain (embryonic critical period: E12 to E18; Perinatal critical period: P0-P10; late postnatal critical period: P10-P40). It summarizes the events that occur during brain maturation and leads to long-term alterations. OD, ocular dominance; ENOs, early network oscillations; GDP, giant depolarizing potentials; GABA, switch from excitatory to inhibitory.

Before experience-driven critical periods sculpt neural wiring in the postnatal brain, exposure to cytotoxic agents has been used to identify timepoints during which cell proliferation, migration, differentiation and apoptosis are susceptible to change (for review Rice and Barone, 2000; Figure 2). Recent studies have unveiled new molecular mechanisms acting in immature cell types during neurogenesis and programmed cell death. New evidences suggest that neurogenic fate and programmed cell death might be influenced by their surrounding milieu as well as by electrical activity (Blanquie et al., 2017; Vitali et al., 2018; Wong et al., 2018; Oberst et al., 2019). In the following section, we highlight recently discovered features that characterize developmental plasticity events in neurogenesis and apoptosis and that extend beyond previous criteria that define a classical critical period (for review Hensch, 2004).

**Figure 3:**
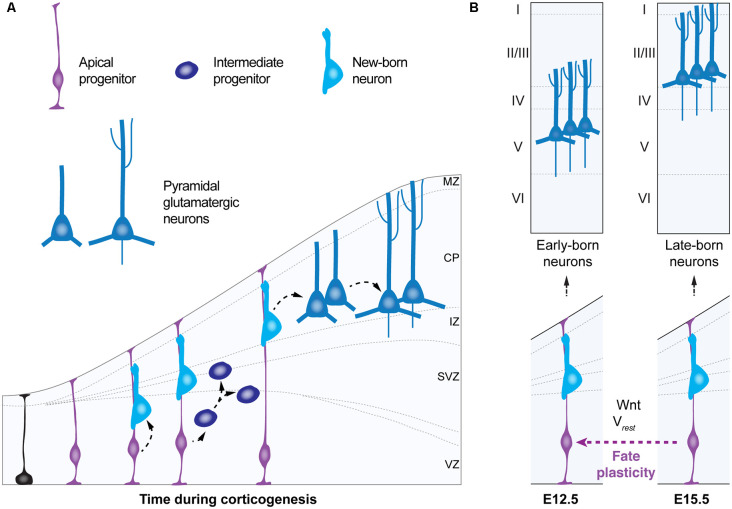
Fate plasticity during corticogenesis. **(A)** Schematic of corticogenesis (left panel) showing three populations of progenitor cells: neuroepithelial cells (in black), apical progenitors (light blue) and intermediate progenitors (dark blue). Apical progenitors (radial glia) within the ventricular zone (VZ) initially expand their population and generate neurons and intermediate progenitors. As corticogenesis proceeds, intermediate progenitors (within the SVZ) as well as apical progenitors generate neurons. **(B)** Temporal progression of neurogenic stage (right panel) shows apical progenitors around embryonic stage (E) 12.5 that give rise to early born deep-layer pyramidal neurons. In the standard temporal progression of corticogenesis apical progenitors at E15.5 give rise to late-born upper-layer pyramidal neurons. Apical progenitors at E15.5 can change their neurogenic state, a process named “fate plasticity” and give rise to late-born deep-layer pyramidal neurons under specific conditions such as cell hyperpolarization or reactivation of Wnt-signaling. VZ, ventricular zone; SVZ, subventricular zone; IZ, intermediate zone; CP, cortical plate; MZ, marginal zone; V_rest_, resting membrane potential.

During corticogenesis in the embryonic brain, apical progenitors give rise to pyramidal neurons in an “inside-out” fashion—first deep, later superficial (Cadwell et al., 2019). Intrinsically-regulated genetic programs in apical progenitors guide their temporal progression in neurogenic fate from early states—in which they generate early-born deep layer pyramidal neurons—to a late state—in which they give rise to late-born upper layer pyramidal cells (Figure 3; Telley et al., 2016). Multiple findings from loss-of-function approaches of specific transcription factors suggest that apical progenitors are competent to re-enter a previous neurogenic state, in other words, to rewind their temporal neurogenic state, i.e., to convert into a different progenitor type, a process known as “*fate plasticity*” (Figure 3). For example, the transcription factor *Foxg1* has been shown to regulate at the embryonic stage (E)13.5, the ability of apical progenitors to revert to an earlier neurogenic competence and to generate early-born Cajal-Retzius cells, instead of deep-layer pyramidal neurons (Hanashima et al., 2004; Shen et al., 2006). Interestingly, recent studies support the idea that fate plasticity might be regulated not only by intrinsic genetic programs (for review Greig et al., 2013), but by the bioelectric membrane properties as well as in response to cell-extrinsic factors, in a non-cell autonomous manner (Vitali et al., 2018; Figure 3). Fate plasticity is also specific for different progenitor types and specific of a time point in corticogenesis. In contrast to intermediate progenitors—displaying an immutable temporal progression of neurogenic fate—apical progenitors at embryonic stage (E)15.5 can re-enter a past molecular state—from E15.5 neurogenic state to E12.5—giving rise to additional deep-layer neurons within their progeny (Oberst et al., 2019). Thus, fate plasticity of apical progenitors could meet several principles that define a critical period (for review Hensch, 2004). The first principle is the influence by the environment. It has been shown that fate plasticity is dependent on the resting membrane potential of radial glia (Vitali et al., 2018), their metabolic states (Khacho et al., 2016; Knobloch et al., 2017; for review Knobloch and Jessberger, 2017) and the feedback signaling from neighboring postmitotic neurons (Seuntjens et al., 2009; Toma et al., 2014). This suggests that fate plasticity could be influenced by surrounding factors regulating apical progenitor’s microenvironment, such as the availability and distribution of ions and metabolites that constitute the electrochemical gradient and determine the energetic states of these cells. A second principle met by fate plasticity of apical progenitors is its occurrence during a unique time window in embryonic development: apical progenitors are a transient cell type that displays fate plasticity at embryonic stage (E) 15.5. A third principle of a critical period is the irreversible effects on brain function. Although fate plasticity is a process that will generate higher numbers of deep-layer cortical neurons (i.e layers 5–6), instead of upper-layer ones (i.e., layers 1–4) irreversibly, the precise consequences of fate plasticity for adult brain function remain to be elucidated. In addition, since the generation of astrocytes and oligodendrocytes follows neurogenesis and continues during the first postnatal month in the mouse brain (Bayraktar et al., 2014; for review Bergles and Richardson, 2015), an unanswered question is whether fate plasticity of radial progenitors could also impact the production of non-neuronal cells in the cerebral cortex. It is easy to speculate that if the generation of high numbers of deep-layer pyramidal neurons occurs at the expense of upper-layers neurons and glia, fate plasticity could have a significant impact on cortical computation and on the regulation of subsequent critical periods.

Programmed cell death in the postnatal brain is also a critical period for network composition. The immature brain generates a surplus of cells necessary to guide specific processes during development. Intrinsic molecular programs initiated by the pro-apoptotic factors of Bcl2-associated X protein (Bax) and Bcl2 antagonist/killer (Bak) render specific cell populations susceptible to undergo cell death during brain development (Southwell et al., 2012). Brain cells undergo massive elimination during two waves of apoptosis that take place from the embryonic stage E14 and between P6 and P10 in mice (Figure 2; for review Wong and Marin, 2019). In contrast to previously reported cell-autonomous regulation of cell death (Southwell et al., 2012), new evidence suggests that specific patterns of postnatal network activity establish the onset, duration and extent of neuronal apoptosis (Blanquie et al., 2017; Denaxa et al., 2018; Priya et al., 2018; Wong et al., 2018; Duan et al., 2020). Recent work identified two molecular pathways guiding the selection process that determines neuron survival and sets the final composition of a neural network in the cerebral cortex: the calcium-dependent phosphatase Calcineurin, that translates the arrival of electrical activity into molecular cascades of cell survival (Priya et al., 2018), and the phosphatase tensin homolog protein (PTEN), that sets the clock for the death of the GABAergic interneurons in the absence of incoming activity from pyramidal neurons (Wong et al., 2018). Neuronal numbers significantly decrease during the first two postnatal weeks, reaching a plateau in adulthood in the neocortex (Blanquie et al., 2017; Denaxa et al., 2018; Priya et al., 2018; Wong et al., 2018). As apoptotic neurons cannot be replaced, programmed cell death in the postnatal brain is an irreversible process. The main feature that differentiates programmed cell death from classical critical periods is the functional consequence of postnatal apoptosis for cortical network response. In classical critical periods, the functional competence of inputs determines the selection of a specific pattern of connectivity. Yet, during programmed cell death, the functional competence of inputs determines the final number of neurons and the relative contribution of GABAergic and glutamatergic neuronal types to the neural network. Therefore, it is conceivable to believe that changes in neuronal number (through “*critical periods for network composition*”) could have a different impact on the dimensionality of neural responses than changes in connectivity patterns (through “*classical critical periods*”). Currently, it is unclear how programmed cell death of postnatal neurons precisely influences information processing in the cerebral cortex. However, initial studies performed in mice demonstrate that manipulating the number of excitatory pyramidal neurons in the cerebral cortex results in permanent consequences for brain function and behavior. Glutamatergic neuron overproduction as well as increased glutamatergic neuron apoptosis result in altered motor learning, hyperactivity or phenotypes reminiscent of autism-like traits (e.g., altered social behavior and repetitive behavior; (Fang et al., 2014; Nakamura et al., 2016). Moreover, programmed cell death significantly changes the size of the visual cortex leading to smaller, but functional, retinotopic map (Nakamura et al., 2016). These findings suggest that programmed cell death during postnatal stages is a critical period for high-order complex behavior such as social behavior, and motor learning. Further studies should unveil whether programmed cell death in postmitotic glutamatergic neurons is a critical period for sensory (e.g., visual) function.

## Manipulating Critical Periods to Shift Developmental Trajectories

In the context of disease, knowledge of critical period plasticity shows promise to restore specific aspects of brain function in animal models of neurological disorders. Control of critical period plasticity can be implemented in two different ways: by reopening critical periods in the adult brain—which is the most common strategy—and by prematurely closing the periods of abnormal plasticity through timely intervention in the early developing brain, requiring more sophisticated experimental designs. In the following section, we highlight recent pre-clinical studies that illustrate the therapeutic potential of these two timely strategies targeting developmental plasticity specific to critical periods.

The reactivation of restricted periods of plasticity in adulthood represents both an episode of vulnerability and opportunity for therapeutic intervention (for review Hensch and Bilimoria, 2012; Marín, 2016). The main advantage of thoroughly understanding critical periods is to be able to manipulate their onset, duration, and closure to ultimately control heightened levels of brain plasticity. The identification of molecular mechanisms regulating critical periods in the developing brain has been a key stepping-stone towards unveiling approaches that control the reopening of heightened plasticity after closure of the developmental plasticity period. Numerous approaches through which critical windows—and thus brain malleability—might be re-opened in adults have been attempted in animal models. Molecular factors, such as the endogenous inhibitor for nicotinic acetylcholine receptor—Lynx1—and other components of the extracellular matrix, i.e., perineuronal nets (PNNs), set the closure of OD plasticity and limit plasticity in the adult visual cortex (Pizzorusso et al., 2002; Morishita et al., 2010; for review Hensch, 2005; Fawcett et al., 2019; Yang, 2020). These factors are key for the maintenance of network activity (Favuzzi et al., 2017; Faini et al., 2018) and brain plasticity (Bradshaw et al., 2018). Moreover, these molecules not only regulate critical periods in sensory function, they also participate in the regulation of critical periods related to complex cognitive demands such as the storage of emotional memories (for review Nabel and Morishita, 2013). In particular, PNNs mediate the closure of critical period of permanent fear memory extinction (Xue et al., 2014; Slaker et al., 2015). Removal of PNNs resets the mature neural network to an immature, juvenile state, by decreasing network inhibition and increased gamma activity (Lensjø et al., 2017). Control of critical period plasticity through PNN regulation in other brain regions influences brain function beyond sensory-motor and complex behaviors. In animal models of obesity and diabetes, critical period for leptin-dependent development of the hypothalamic acuate nucleus—involved in metabolic homeostasis—is accompanied by a reorganization of PNNs around leptin-receptor positive GABAergic neurons (Mirzadeh et al., 2019). Thus, PNNs remain central molecular hallmarks in the study of critical periods associated to the pathophysiology and the treatment of a range of neuropsychiatric disorders and related metabolic comorbidities (Bradshaw et al., 2018; for review Wen et al., 2018).

Other approaches took advantage of molecules with a synaptic function that participate to critical periods of synaptic plasticity during a specific time window for social reward learning. In particular, oxytocin-mediated long-term depression in the nucleus accumbens is reopened by a single dose of 3,4-Methyl enedioxy methamphetamine in young adult mice (MDMA, commonly known as ecstasy; (Nardou et al., 2019). Consequently, activation of the oxytocin receptors by MDMA showed potential to restore impaired social behavior and social-related disorders in animal models of neurodevelopmental disease. Other studies reported that drug-induced reopening of critical period plasticity specifically improves the function of sensory systems. For example, valproic acid—an histone deacetylase inhibitor affecting synaptic neurotransmission and primarily used to treat epilepsy—has been shown to restore visual acuity in animals that underwent monocular deprivation (Silingardi et al., 2010). Valproic acid has also been shown to participate to the reopening of the critical-period learning of absolute pitch in humans (Gervain et al., 2013).

Additional experimental strategies involve the direct manipulation of inhibitory GABAergic circuits. Inhibitory interneurons are central arbiters of critical period plasticity and direct the competition between electrical activity and patterns of connectivity setting plasticity onset and duration (Toyoizumi et al., 2013; Tang et al., 2014; Isstas et al., 2017). Specific subpopulations of inhibitory interneurons define the tempo of experience-dependent critical period in the cerebral cortex (for review Hensch, 2005). Fast-spiking PV-cells have the potential to remain plastic even beyond the peak of natural critical periods (Morishita et al., 2015). The transient increase of parvalbumin interneuron activity with pharmacological approaches (diazepam) or with approaches that employ synaptogenic molecules to increase synaptic excitatory input onto PV cells—through exogenous application of Neuregulin1, a ligand of the interneuron-specific transmembrane receptor ErbB4—prevent OD plasticity after visual monocular deprivation (Kuhlman et al., 2013; Sun et al., 2016). Pharmacological or chemogenetic approaches that increase PV interneuron activity have also been shown to restore control-like neural activity (CA1 network dynamics) and behavior (cognitive function) in different mouse lines modeling mutations associated to schizophrenia (Marissal et al., 2018; Mukherjee et al., 2019).

Failure to stabilize neural circuits and reducing plasticity as the brain develops underlies the physiopathology of a range of neurodevelopmental disorders. One example is delayed critical period for GABA polarity in the FXS mouse model (also known as *Fmr1* deficient mice; He et al., 2014) and in mouse models of Rett syndrome (*Mecp2* deficient mice) displaying abnormal GABA polarity in the hippocampus (excitatory GABA instead of inhibitory), until very late in brain development (i.e., P15 in mice (Lozovaya et al., 2019). In addition, *Fmr1* deficient mouse models of FXS are also characterized by a delayed progression of critical periods in the somatosensory system (Bureau et al., 2008; Harlow et al., 2010; Till et al., 2012). Interestingly, precocious onset and closure of critical period in the visual cortex has been reported in *Mecp2* deficient mice (Durand et al., 2012; Krishnan et al., 2015). In order to prematurely advance, constrain or delay critical periods of plasticity, acute experimental strategies have been employed to reorient abnormal immature brain states towards a normal developmental trajectory. One of these strategies took advantage of timely manipulations of GABA action during early postnatal development or around birth, through the inhibition of the Na^+^/K^+^ Cl^−^ cotransporter 1 (with NKCC1 inhibitors, i.e., bumetanide). These efforts normalized somatosensory whisker-evoked responses and GABA polarity in the mouse hippocampus of the FXS mouse model (He et al., 2019). Altogether these recent findings illustrate how a better knowledge on critical period regulation could be employed (e.g., by re-opening or tuning the degree of plasticity) to develop timely applied strategies that lessen disease-relevant symptoms.

## Modeling Critical Periods of Plasticity with New Technologies

Mouse genetics are a fundamental asset to study the influence of gene-environment relationship on critical periods (Xu et al., 2019). They still remain a key tool to probe the genetic, cellular and neural circuits basis of pathophysiological states of human neurodevelopmental disorders (for review Marín, 2016; Del Pino et al., 2018). The advent of CRISPR-Cas9 mediated gene editing technology has significantly increased the spectrum of animal models available for the study of neurodevelopmental disease, using either rodents but, also recently, non-human primates. A recent study revealed that CRISPR-designed macaque monkeys, mutant for the *Shank3* gene, exhibited sleep disturbances, motor deficits and increased repetitive behavior, as well as social and learning impairments (Zhou et al., 2019). Similarly, lentivirus-mediated transgenic monkeys expressing human MeCP2 in the brain exhibit autistic-like behavior and show germline transmission of the transgene (Liu et al., 2016). Whilst the use of non-human primates as animal models provides some scientific benefits to further unveil critical period plasticity mechanisms underpinning human disorder-like pathophysiology, we focus here on cutting-edge technological advances that will importantly contribute to model critical periods of the human brain.

Reconstructing the developmental trajectories of human cortical circuits *in vitro* has the potential to revolutionize our understanding of vulnerable periods in human brain development (Chukwurah et al., 2019; Marshall and Mason, 2019; for review Quadrato et al., 2016). In order to fill the gap between human disease and model organisms, the development of stem cell technologies—both embryonic and induced pluripotent stem cells (iPSCs)—have given access to functional readouts typical of early stages of human brain development (Dolmetsch and Geschwind, 2011). iPSCs have provided insights into the cellular alterations underlying neuropsychiatric disorders such as autism and schizophrenia (Marchetto et al., 2017; Adhya et al., 2020; reviewed in Ben-Reuven and Reiner, 2016; Vitrac and Cloëz-Tayarani, 2018). Although concerns have been raised regarding the clinical applications of iPSCs as a valuable source for cell transplantation therapy and the significant reprogramming variability of human-derived iPSCs, the simplicity of two-dimensional cultures is suitable for mechanistic studies, large-scale screening, or high-throughput drug testing. However, modeling critical period *in vitro* requires a better system that recapitulates the processes of proliferation, patterning, cell fate progression, migration and connectivity rearrangement of neural networks composed of distinct cell types and organized into different layers and different systems. It also demands an approach that will ultimately include environmental cues influencing different levels of plasticity during protracted periods of time.

**Figure 4:**
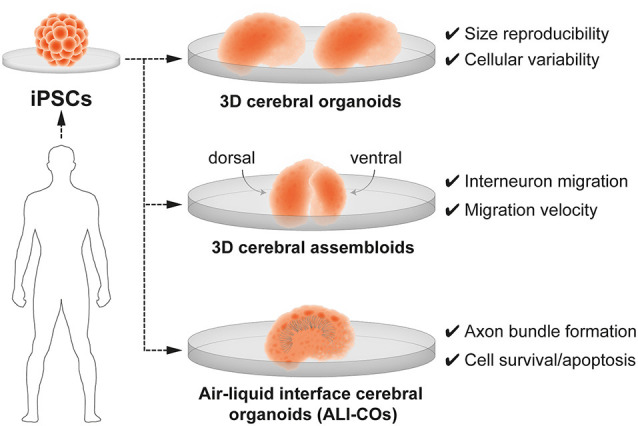
3D brain organoids for modeling human brain development and neurodevelopmental disorders *in vitro*. Cerebral organoids are 3D brain models derived from human induced-pluripotent stem cells (iPSCs). 3-D brain organoids generated by Velasco et al. (2019; top panel) are consistent in size and reproducibly present a spectrum of cell types. 3-D cerebral assembloids (middle panel) are formed of a pallium-like organoid (dorsal) and a subpallium-like organoid (ventral) and can recapitulate GABAergic interneuron migration. Air-liquid interface cerebral organoids (ALI-Cos; bottom panel) show an improved neural survival and can therefore be suitable to assay critical periods of programmed cell death. Neurons within ALI-COs form axon bundles over long-distances mimicking long-range projections.

The generation of three-dimensional (3-D) brain organoids, derived from human induced pluripotent stem cells (iPSCs), resolved the long-standing limitations of a 2D approach and provide unprecedented opportunity to understand neurobiological mechanisms of human brain development in health and disease. Currently, 3D brain organoids have a demonstrated validity to model basic features of the human embryonic neural tissue and critical steps of early brain development such as neurogenesis, neuronal migration and neuroanatomical features—upper and deep layer neurons—specific of brain tissue (reviewed in Paşca, 2018; Seto and Eiraku, 2019; Benito-Kwiecinski and Lancaster, 2020; Tambalo and Lodato, 2020; Velasco et al., 2020). Important advances in the engineering of brain organoids have also resolved long-standing limitations in size reproducibility and in the production of neuronal diversity which closely resembles the one found in *in vivo* embryogenesis (Velasco et al., 2019). Nevertheless, 3D brain organoids present some limitations, namely the inability to reproduce the exact cell type diversity and radial glia maturation (Bhaduri et al., 2020). It also remains unclear whether fate plasticity in cortical radial glia can be probed in brain organoids. Despite these constraints, brain organoids are suitable to replicate some developmental processes of the human brain such as the transcriptional regulation of neural progenitor cell fate, altered in pathology such as ASD (Mariani et al., 2015). Moreover, brain organoids composed of iPSC derived from pallial (cortical) and sub-pallial (subcortical) domains, also named 3-D cerebral assembloids, have been utilized to model tangential migration of interneurons (Bagley et al., 2017; Birey et al., 2017; Xiang et al., 2017) and reciprocal cortico-thalamic connectivity (Xiang et al., 2019; Figure 4). Interestingly, cerebral assembloids derived from cells of patients with Timothy syndrome—a severe neurodevelopmental disease characterized by ASD and epilepsy—were used to demonstrate that GABAergic interneurons exhibit prominent migratory defects (Birey et al., 2017).

In order to bypass limitations of brain organoids in neuronal survival and axonal growth, further efforts implemented new culturing methods, such as the air-liquid interface cerebral organoids (ALI-COs). ALI-COs have been useful in the generation of brain organoids with specific features more reminiscent of *in vivo* neural networks, such as the formation of functional axonal bundles spanning long-range—longer than in other brain organoids—projections (Giandomenico et al., 2019). It remains to be determined to which extent 3D brain organoids, ALI-Cos or 3-D cerebral assembloids can recapitulate the key periods of fate plasticity, embryonic and postnatal programmed cell death, intrinsic network activity, as well as input-dependent network plasticity. Neuron-glia interactions and presence or absence of mesoderm-derived progenitors giving rise to microglia represent sources of variability in these preparations (Ormel et al., 2018). Therefore, touching upon the role of glia when modeling vulnerable windows of plasticity in brain organoids is very complex and should be the subject of discussion of experts in the field.

Current challenges of the *in vitro* brain organoid systems to model critical periods require further systematic efforts to reliable model the emergence of network activity patterns and new approaches that mimic environmental influences together with techniques tailored to measure their long-term consequences in organoid culture conditions. So far, brain organoid preparations display spontaneous neural activity (Eiraku et al., 2008; Quadrato et al., 2017; Giandomenico et al., 2019). Mouse-derived brain organoids display robust network activity synchronization resembling an immature form of functional organization (Eiraku et al., 2008), but neither it has been shown whether human-derived brain organoids consistently display synchronized network activity within the same type of preparations (Quadrato et al., 2017) nor it is known whether they recapitulate the sequence of coordinate network activity observed *in vivo* (Giandomenico et al., 2019). It was suggested that brain organoid activity could be tailored to a specific activity pattern using optogenetics (Velasco et al., 2020). Imposing a specific activity pattern to brain organoids could be indeed useful to better understand the logic behind the interplay between electrical activity and molecular programs specific to human brain development. Nevertheless, an in-depth characterization of network activity patterns intrinsically displayed by brain organoids across time needs to be performed. For example, recent studies showed robust synchronized network activity in human-derived brain organoid cells, when cells were dissociated and plated anew (Sakaguchi et al., 2019) and giant-depolarization potential-like events are displayed by a newly developed type of neuronal organoid preparation known as Bioengineered Neuronal Organoid (BENO; Zafeiriou et al., 2020). Yet, comparative molecular profiling and connectivity rules of highly synchronized brain organoids vs. non-synchronized ones will further elucidate neurobiological aspects supporting early patterns of intrinsic network activity in the human brain. The field will profit from a standardization of the cellular components and environmental cues (culturing conditions) necessary for brain organoids to display the synchronized network activity patterns found *in vivo*.

In addition, some aspects of the environmental cues influencing early critical periods could be easily probed in human embryonic stem cell-derived brain organoids. Human-derived neurons within BENOs display the developmental switch in GABA polarity found in the developing mouse brain (Zafeiriou et al., 2020). Regulation of critical period for GABA polarity by experience in form of hormonal stimuli could be easily modeled and should be tested through the addition of different concentrations of oxytocin to BENOs during the time period in which GABA is excitatory.

Modeling critical periods of sensory systems typically occurring during early childhood (or early adult mice) in a dish faces many technical challenges and will probably require of brain assembloid technology. Brain organoid preparations containing photoreceptor-like cells and forebrain-like structures, display neural activity in response to light stimuli (Quadrato et al., 2017). The advent of sophisticated experimental designs that combine multiple region-specific organoids together with optogenetic stimulation is necessary to make progress on this front. Exciting times in which basic aspects of cortico-thalamic plasticity can be deconstructed into assembloids containing multiples organoids—e.g., future retina-thalamic-cortical assembloids or bi-thalamic-cortical assembloids—aided by a focal optogenetic stimulation (or controlled environmental conditions) is one of many speculative examples that could help to better assess gene/environment interactions during precise developmental periods. This will unveil if 3D brain organoid/assembloids can recapitulate cellular and molecular hallmarks of classical critical periods, which will ultimately determine the degree of validity of this *in vitro* system as model of human brain development.

## Discussion

Critical periods hold potential to reinstate brain function. We provide a perspective on how the different aspects of critical period research paves the way for potential disease-modifying therapeutic strategies in neurodevelopmental disorders. Timely interventions during specific developmental windows restore network activity or behavior in animal models, thus reinforcing the notion that critical episodes of developmental plasticity can be used as unique windows of therapeutic opportunity to reorient pathophysiological states towards a “normal” developmental path (Marín, 2016). Critical period research raises important considerations for the well-timed administration of therapeutic strategies during precise developmental windows. The goal of timely applied interventions is to reduce undesired trade-off effects of therapeutic strategies utilized during a protracted period within brain development or after brain maturation. Only a sound understanding of the order, time-line and cross-regulations of critical periods in normal and pathological trajectories will help us find the most precise, effective and age-specific therapeutic intervention to follow. With the hope that interventions during specific time windows could significantly help to reduce symptoms in adulthood, further efforts to unveil molecules that reopen or close critical period plasticity will definitely expand the potential clinical relevance of timely interventions during development.

A key challenge to further understand the time-course of vulnerable periods during brain formation is the study of sex-specific differences. A wealth of data demonstrates that the critical period of sexual differentiation in the brain starts in the embryonic brain (from E16 to P4 in mice and second trimester in human; McCarthy et al., 2018). However, there is still very limited information regarding how it influences the time course of subsequent critical periods in males compared to females. Therefore, differentiating sex-specific developmental trajectories represents a major milestone that must be addressed. It is not expected that the timeline of critical periods would be shifted in very basic sensory modalities. However, one could expect that the temporal unfolding of vulnerable periods that determine a different degree of performance of high-order functions to be singular for each sex. Descriptions of sexual differences in brain function show that the temporal extent of sensitive periods for social learning is delayed in males, compared to females (Nardou et al., 2019). The underlying neurobiological basis remains largely underexplored, e.g., the influence of the thyroid gland, expressing both estrogen and androgen receptors on vulnerable periods (Batista and Hensch, 2019). Understanding how hormones impact vulnerable periods in a sex-specific manner is the first step towards the development of personalized medicine. More basic research on gender-specific neurodevelopment is also expected to precisely unveil predisposition and susceptibility of each sex to brain disease, within a framework that encompasses the life-experience of the individual.

Finally, careful classification of vulnerable periods as “critical” or “sensitive” has implications beyond basic research. Policymakers, psychosocial therapies and educational aid programs for early childhood adversity should be guided by the scientific advances in critical period research (see recent review Nelson and Gabard-Durnam, 2020). Specially now, in light of the current challenges posed by the COVID19 pandemic, measures of confinement and social distancing on millions of healthy children and children with neurodevelopmental disorders are lacking a consensus feedback from the scientific community (Arango, 2020). This is mirrored by a worrisome scenario in which governments apply very different measures that raised skepticism and questions such as: How long could we implement confinement measures while maintaining neuropsychiatric well-being in each age-group of children? Are the potential long-term consequences of confinement in children with neurodevelopmental disorders—such as ASD—exceeding the risk of COVID19 infection? (Aledo-Serrano et al., 2020). A better understanding of critical periods as opposed to sensitive periods is fundamental to instruct these policies, considering the implications for mental health of the next generations.

## Author Contributions

ND and ID contributed equally.

## Conflict of Interest

The authors declare that the research was conducted in the absence of any commercial or financial relationships that could be construed as a potential conflict of interest.
